# Detection of *bla*_CTX-M15_ and *bla*_OXA-48_ genes in Gram-negative isolates from neonatal sepsis in central of Iran

**Published:** 2019-08

**Authors:** Taiebeh Shakiba, Alireza Sadeghnia, Vajihe Karbasizade

**Affiliations:** 1 Department of Microbiology, School of Medicine, Isfahan University of Medical Sciences, Isfahan, Iran; 2 Department of Paediatrics, School of Medicine, Imam Hossein Hospital, Isfahan University of Medical Sciences, Isfahan, Iran

**Keywords:** Neonatal sepsis, Drug resistance, *bla*_CTX-M-15_ gene, *bla*_OXA-48_ gene

## Abstract

**Background and Objectives:**

The aim of this study was to determine the prevalence of neonatal sepsis with a focus on antibiotic resistance and the frequency of the *bla*_CTX-M-15_ and *bla*_OXA-48_ genes in Gram-negative isolates.

**Materials and Methods:**

A total of 108 Umbilical Cord Blood (UCB) and 153 peripheral blood samples were cultured via BACTEC from May 2017 to June 2018. The bacterial isolates were identified using phenotypic and genotypic analyses. The antibiotic susceptibility profile of the isolates was determined by disk diffusion. PCR was used to determine the frequency of β-lactamase genes.

**Results:**

Among the 153 infants, 21 (13.7%) proved positive for sepsis. *Escherichia coli, Staphylococcus epidermidis* and *Klebsiella pneumoniae* were the most frequent isolates in the peripheral blood cultures. *E. coli* and *Stenotrophomonas maltophilia* were isolated from two UCB cultures. The highest resistance among the Gram-positive strains was to cefixime, ceftriaxone, cefotaxime and clindamycin. In the Gram-negative bacteria the highest rates of resistance were to ampicillin (91.7%). The frequency of *bla*_OXA-48_ and *bla*_CTX-M-15_ genes was 25% and 50%, respectively.

**Conclusion:**

The high antibiotic resistance among the isolates reveals the importance of monitoring antibiotic consumption and improving control standards in the health care system, especially in neonatal wards.

## INTRODUCTION

Neonatal sepsis refers to a bacterial bloodstream infection presenting with systemic symptoms that is classified into two groups, including Early-Onset Sepsis (EOS) and Late-Onset Sepsis (LOS). EOS is usually caused by vertical transmission and appears during the first three days after childbirth, while horizontal transmission by hand/body contact or intra-venous catheters can lead to LOS within four to 28 days of birth (1). Despite the outstanding advances in neonatal medicine, sepsis remains a leading cause of death, and approximately five million newborns die around the world every year as a result of these infections (2). The etiologic agents of neonatal sepsis vary widely across the world; however, according to reports, Gram-negative bacteria are of greater importance in developing countries and Group B *Streptococci* (GBS) in developed countries (3). The use of antibiotics, especially in developing countries, has resulted in the emergence of antibiotic resistance in Gram-negative bacterial agents and has posed a serious challenge for the treatment of this infection.

Gram-negative bacteria exhibit resistance to β-lactam antibiotics by producing β-lactamase, including Extended-Spectrum β-lactamases (ESBLs) and Oxacillin-hydrolyzing β-lactamase (4). Today, among the existing ESBLs, CTX-M-Type β-lactamases are widely spread in Gram-negative bacteria and have caused their resistance to antibiotics such as cefotaxime, ceftriaxone and ceftazidime (5). Certain OXA-Type enzymes, such as *bla*_OXA-48_, have been of great interest in recent years owing to their ability to hydrolyze carbapenems and spread extensively among *Enterobacteriaceae* strains (6). Moreover, the strains that produce this enzyme also show a secondary non-enzymatic resistance mechanism (7). Given all the noted issues, the empiric antimicrobial therapy for sepsis in developing countries requires sufficient knowledge about common bacterial agents and their antibiotic resistance in every geographical location, so that, in addition to overcoming the challenge of treatment, the emergence and spread of resistance through the inappropriate use of antibiotics can also be prevented in hospital settings.

The present study was designed to assess the frequency and origin of neonatal infection and determine the antibiotic resistance pattern with an emphasis on the frequency of *bla*_CTX-M-15_ (Cefotaximase-M-15) and *bla*_OXA-48_ (Oxacillinase-48) resistant genes in Gram-negative isolates at Shahid Beheshti-hospital in Isfahan, Iran.

## MATERIALS AND METHODS

### Samples and patients.

From May 2017 to June 2018, UCB samples were collected from neonates at a teaching hospital of Isfahan, who met at least two of the following criteria: Premature birth (less than 37 weeks), prolonged rupture of the membranes (18 hours), prolonged delivery (more than 24 hours) and multiple vaginal examinations. Peripheral blood samples (PB) of the newborns with even one symptom of sepsis were also examined. A total of 153 peripheral blood samples and 108 UCB samples were collected from the neonates. Three different peripheral blood samples were taken from each neonate. The first sample was taken at the onset of fever, the second one was taken 60 min later and the third one 24 h later.

At first, all the samples were cultured in BACTEC medium (FX40 BD, USA). They were then cultured on blood agar (5% sheep blood), eosin methylene blue (EMB) and chocolate agar. The plates were incubated at 37°C with 5% CO_2_ for 24–48 h.

### Characterization assays.

The Gram-positive isolates were identified using biochemical tests such as the catalase test, the coagulase test, Novobiocin susceptibility assay, arabinose fermentation, the bile esculin test and the CAMP test, while the IMVIC, urea, TSI, SIM, LIA and OF tests and oxidase fermentation were used for evaluating the Gram-negative strains (8). 16S rRNA was amplified using the universal primers RWO1 and DG74 for the identification of all the isolate (9). Thermal cycling consisted of initial denaturation at 95°C for 2 minutes followed by 5 cycles of denaturation at 94°C for 1 minute, annealing at 60°C for 50 s, extension at 72°C for 1 minute and 38 cycles of denaturation at 94°C for 1 minute, annealing at 58.5°C for 1 minute, and extension at 72°C for 1 minute.

### Antibiotic susceptibility testing.

The antibiotic resistance assay was performed according to the Clinical and Laboratory Standards Institute (CLSI) guidelines using the agar disc diffusion method (DDM) for vancomycin (30 mg), ampicillin (10 mg), ciprofloxacin (5 mg), ceftriaxone (30 mg), meropenem (10 mg), colistin (10 mg), cefoxitin (30 mg), cefotaxime (30 mg), cefixime (5 mg), amoxy clavulanic acid (20/10 mg), gentamicin (10 mg), erythromycin (30 mg), clindamycin (2 mg) and penicillin (10 mg) [MAST, UK] ;(10). The E test was used for evaluating the susceptibility to vancomycin and colistin.

### Detection of the beta-lactam resistance genes.

The isolates were first evaluated by the Modified Hodge Test (MHT), and those that showed positive results in this test were used for the detection of *bla*_OXA-48_ and *bla*_CTX-M-15_ resistant genes with previously-published PCR primers (11). DNA was extracted from all the isolates by a SinaPure TM DNA kit (Cinagen, Tehran, Iran) according to the manufacturer’s protocol. To detect the *bla*_CTX-M-15_ gene, the PCR was performed as follows: Initial denaturation at 95°C for 2 min, 35 cycles of 94°C for 1 min, 52°C for 1 min, 72°C for 90 s and a final extension at 72°C for 3 min. The *bla*_OXA-48_ gene was amplified according to the following conditions: Initial denaturation at 95°C for 5 min, followed by 35 cycles of denaturation at 95°C for 30 s, annealing at 54°C for 40 s, extension at 72°C for 45 s and a final extension at 72°C for 5 min.

### Statistical analysis.

The measurements were reported as mean ± standard deviation (SD) for the quantitative variables and percentage or rate for the categorical variables. Inter-group comparisons were conducted in SPSS-22 using the χ^2^ test and Fisher’s exact test. P<0.05 was regarded as statistically significant.

## RESULTS

### Baseline characteristics.

A total of 153 peripheral blood and 108 UCB samples were investigated during the 13 months of the study. The neonates’ age ranged from 1 to 28 days (mean =4.7±6.9 days). The blood culture results confirmed sepsis in 21 neonates, including nine (43%) boys and 12 (57%) girls, with a 1.3:1 girl/boy ratio. The youngest neonate was a day old and the oldest 20 days old. Table 1 presents the results of the umbilical cord and peripheral blood cultures by EOS and LOS. Out of the 21 sepsis cases, 71% were LOS and 29% were EOS. The umbilical cord blood culture result was not positive in the neonates with LOS.

**Table 1.** The results of the umbilical cord and peripheral blood cultures by EOS and LOS

	**Result**	**EOS (n=6)**	**LOS (n=15)**	**P-value**
**CRP**	Neg.	50%	0	0.02
	Pos.	50%	100%	
**WBC**	Leukopenia	33.3%	0	0.02
	Normal	66.7%	73.3%	
	Leukocytosis	0	26.7%	
**Platelet**	Normal	60%	64.3%	0.23
	Thrombocytosis	20%	0	
	Thrombocytopenia	20%	35.7%	
**Gender**	Male	33.3%	46.7%	0.58
	Female	66.7%	53.3%	

### Blood tests.

Table 2 presents the diagnostic laboratory parameters by EOS and LOS. Leukocyte counts <4000 μL were taken as cases of leukopenia, and >20,000 μL leukocytosis cases with a platelet count <150,000 were taken as indicative of thrombocytopenia and >450,000 as indicative of thrombocytosis. C - reactive protein (CRP) levels lower than or equal to 6 were assumed to be negative. Fisher’s exact test revealed a significant correlation between positive CRP levels and LOS, since the CRP level of the neonates with LOS was higher than that of the neonates with EOS (P=0.02). The χ^2^ test showed that leukopenia is significantly higher in neonates with EOS, while leukocytosis was higher among the LOS neonates (P=0.02). The χ^2^ test also demonstrated no relationship between the frequency distribution of gender and the prevalence of LOS or EOS (P=0.58).

**Table 2.** Early-onset sepsis (EOS) and late-onset sepsis (LOS) characteristics.

**EOS**	**No (%) (n=153)**	**LOS**	**No (%) (n=153)**
UCBC (+) & PBC (+)	2 (1.3)		7 (4.6)
UCBC (−) & PBC (+)	4 (2.6)	UCBC (NT) & PBC (+)	8 (5.2)
Total	6 (3.9)	UCBC (−) & PBC (+)	15 (9.8)

EOS: Early-onset sepsis, LOS: Late-onset sepsis, UCBC: Umbilical cord blood culture, PBC: peripheral blood culture, NT: Not tested.

A total of 22 bacterial isolates were traced in the neonates’ blood samples, in which *Klebsiella pneumoniae, Staphylococcus epidermidis* and *Escherichia coli* were the most common isolated pathogens. *S. maltophilia* and *E. coli* were isolated from two UCB cultures compatible with the peripheral blood culture results, which shows that the bacteria were transmitted vertically from the mother to the neonate. In the present study, there was only one case of co-infection with *E. coli* and *K. pneumoniae*.

### Antibiotic resistance profile.

**Fig. 1:**
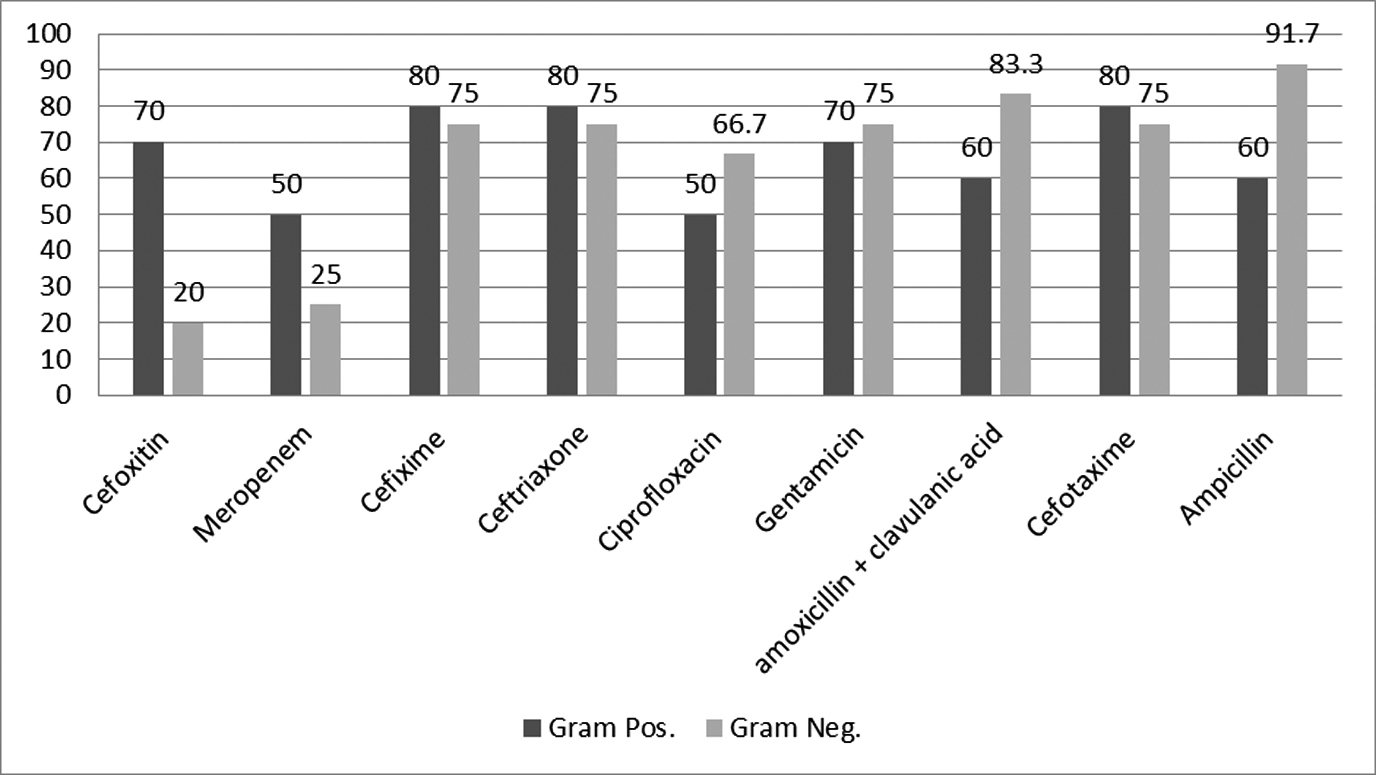
Prevalence of antibiotic resistance among Gram positive and Gram negative isolates from neonatal sepsis.

Fig. 1 presents the antibiotic resistance profile of the Gram-positive and Gram-negative isolates. All the Gram-negative isolates were susceptible to colistin, and the highest resistance was found to ampicillin (91.7%). Regarding the results of the DDM and E test for the Gram-positive isolates, the highest sensitivity to vancomycin was 70% and the greatest resistance to cefixime, ceftriaxone, cefotaxime and clindamycin was 80%. All the *S. epidermidis* strains were susceptible to novobiocin in this study. A significant finding of the present study was the sensitivity of *S. maltophilia* strain to all the tested antimicrobial antibiotic agents.

Fisher’s exact test showed that resistance to cefoxitin (P=0.01) was significantly greater in the Gram-positive than the Gram-negative bacteria; however, no significant differences were observed between the Gram-positive and Gram-negative bacteria in the patterns of resistance to cefixime (P=0.59), ceftriaxone (P=0.59), gentamicin (P=0.58), cefotaxime (P=0.59) and ciprofloxacin (P=0.59).

The χ^2^ test showed that the frequency of resistance to meropenem was significantly greater in the Gram-positive bacteria while resistance to amoxicillin clavulanic acid and ampicillin was significantly greater in the Gram-negative bacteria. Among the 22 isolates recovered from neonatal sepsis, 18 strains (81.8%) were Multi-Drug Resistant (MDR), containing 54.5% Gram-negative and 45.5% Gram-positive isolates. Fisher’s exact test did not show any significant relationships between the frequency of multi-drug resistance and bacterial Gram class (P=0.37).

### PCR amplification of *bla*_OXA48_ and *bla*_CTX-M-15_ genes.

The frequency distribution of *bla*_OXA48_ and *bla*_CTX-M-15_ genes was found to be 25% and 50% in the Gram-negative isolate. As shown in Table 3, *bla*_OXA48_ gene was traced in only three *A. baumannii* isolates, which also showed resistance to meropenem in the phenotypic analysis. The gene *bla*_CTX-M-15_ was detected in the *Enterobacteriaceae* species, which was consistent with their resistance to cefotaxime and ceftriaxone by the DDM method. In addition, the *bla*_CTX-M-15_ gene had the highest frequency among the *K. pneumoniae* isolates. These two genes were not detected among the *S. maltophilia* strains.

**Table 3.** The antibiotic resistance pattern of the bacterial isolates of neonatal sepsis and the frequency distribution of *bla*_CTX-M-15_ and *bla*_OXA-48_ genes in the Gram-negative strains.

**Bacteria isolates**	***bla*_OXA-48_**	***bla_CTX-M15_***	**EOS**	**LOS**	**Antibiotic resistance profile**
*S. agalactiae*	NT	NT	*	-	GM, E, CD
*E. coli*	−	+	*	-	CFM, CRO, CP, AMC, CTX, AMP
*S. maltophilia*	−	−	*	-	-
*E. faecalis*	NT	NT	*	-	FOX, CFM, CRO, GM, CTX, CD
*S. agalactiae*	NT	NT	*	-	CP, GM, E, CD
*S. viridans*	NT	NT	*	-	CFM, CRO, AMC, CTX, P, V
*A. baumannii*	+	−	-	*	FOX, MEM, CFM, CRO, CP, GM, AMC, CTX, AMP
*K. pneumoniae*	−	+	-	*	CFM, CRO, CP, GM, AMC, CTX, AMP
*K. pneumoniae*	−	+	-	*	CFM, CRO, CP, GM, AMC, CTX, AMP
*E. coli*	−	+	-	*	CFM, CRO, GM, AMC, CTX, AMP
*S. epidermidis*	NT	NT	-	*	FOX, CFM, CRO, CTX, E, CD, P, AMP
*K. pneumoniae*	−	+	-	*	CFM, CRO, CP, GM, AMC, CTX, AMP
*K. pneumoniae*	−	+	-	*	CFM, CRO, CP, GM, AMC, CTX, AMP
*E. faecium*	NT	NT	-	*	FOX, MEM, CFM, CRO, CP, GM, AMC, CTX, E, CD, P, AMP, V
*S. epidermidis*	NT	NT	-	*	FOX, MEM, CFM, CRO, CP, GM, AMC, CTX, E, CD, P, AMP
*S. epidermidis*	NT	NT	-	*	FOX, MEM, CFM, CRO, AMC, CTX, P, AMP
*E. faecium*	NT	NT	-	*	FOX, MEM, CFM, CRO, CP, GM, AMC, CTX, E, CD, P, AMP, VA
*E. coli*	−	−	-	*	AMC, AMP
*A. baumannii*	+	−	-	*	FOX, MEM, CFM, CRO, CP, GM, AMC, CTX, AMP
*S. epidermidis*	NT	NT	-	*	FOX, MEM, FM, CRO, CP, GM, AMC, CTX, E, CD, P, AMP
*A. baumannii*	+	−	-	*	FOX, MEM, CFM, CR0, CP, GM, AMC, CTX, AMP
*E. coli*	−	−	-	*	GM, AMP

NT: Not tested;

*: Isolated from EOS or LOS. Fox: cefoxitin; MEM: meropenem; CFM: cefixime; CRO: ceftriaxone; CP: ciprofloxacin; GM: gentamicin; AMC:amoxy clavulanic acid; CTX: cefotaxime E: erythromycin; CD:clindamycin; P: penicillin; AMP: ampicillin; VA: vancomycin

## DISCUSSION

The *bla*_CTX-M-15_ gene is carried in the plasmid and is the most common β-lactamase leading to severe infection in infants (12). In the present study this gene was found in *K. pneumoniae* and *E. coli* isolates. The *bla*_CTX-M-15_ gene is an extended-spectrum beta-lactamase (ESBL) that is increasing in prevalence especially in *Enterobacteriaceae* (13). The prevalence of this gene in infants has been reported as 63.5% by Sebastien et al. which is consistent with the present findings (14). In another study, the *bla*_C-TX-M-15_ gene was identified as the most common ESBL with a prevalence of 81% among the cases of infant septicemia (15). In one study, 1.8% of all carbapenemases were encoded by the *bla*_OXA-48_ gene (16). In the present study, *bla*_OXA-48_ genes were detected in *A. baumannii*. Class-D carbapenemases were commonly detected in *Acinetobacter* spp although *bla*_OXA-48_ has been reported frequently in *K. pneumoniae* and *E. coli* (17). In the present research, only *S. maltophilia* isolate was sensitive to all the antibiotics tested, and the *bla*_CTX-M-15_ and *bla*_OXA-48_ genes were not traced in it. Meanwhile, this bacterium showed multidrug resistance in most studies (18). This difference is probably due to the differences in the geographical location of the study. In the present study, the *bla*_OXA-48_ gene was detected in all the three isolates of *A. baumannii*, which is concerning, since imipenem and meropenem are the medications of choice for the treatment of infections caused by this pathogen (19). Nonetheless, the *bla*_CTX-M-15_ was not traced by PCR and sequencing in any of the *A. baumannii* isolates. In a study conducted in Tehran by Farajzadeh et al. this gene was traced in 40% of the *A. baumannii* isolates (20). The disparity in the results reflects the diversity of the resistance mechanisms employed by this opportunistic pathogen.

The results obtained in this study through the antibiotic resistance assay in neonatal sepsis are consistent with previous reports on the subject. In these studies, Gram-negative isolates showed the highest resistance to ampicillin, cephalosporins and aminoglycosides. Two (1.3%) vancomycin resistant *E. faecium* (VRE) from LOS and one vancomycin resistant *E. faecalis* from EOS were isolated in this study. Rising VRE is a very alarming issue, since it has limited modes of therapy and causes irreparable damage, such as increasing the risk of mortality. In 2014, Shantala et al. reported a case of VRE in neonatal sepsis (21).

Similar to other studies conducted in developing countries, Gram-negative bacilli were the most common isolates in the present study (22). The most frequent isolates were *K. pneumoniae, E. coli* and coagulase negative staphylococci isolates in this research. Behmadi et al. reported coagulase-negative staphylococci were the most prevalent pathogens isolated from blood specimens in LOS and EOS (23). *K. pneumoniae* and coagulase-negative staphylococci were identified as the most common bacterial isolates in the study by Lamia Mohsen et al. (22). In Lamia Mohsen’s research, the frequency of *S. maltophilia* was 0% in EOS and 2% in LOS; in contrast, the present study found similar results for UCB and peripheral blood cultures in the EOS cases. Since newborns’ mothers are hospitalized for a while, the presence of *S. maltophilia* in both the peripheral blood and UCB cultures in EOS may show that the newborn has been infected vertically through the umbilical cord. A study conducted in 1984 reported *S. maltophilia* in neonatal meningitis and conjunctivitis for the first time in India (24). There were two case reports of *S. maltophilia* in EOS from Kolkata, India, both of which were for premature baby girls with a six-hour membrane rupture during their delivery (25). In a case report by Laxman Basny in 2015, *S. maltophilia* was detected as the causative agent of neonatal sepsis (26). *S. maltophilia* attaches easily to plastic surfaces and forms a biofilm that can be the reason for its high prevalence in hospitals (27).

Sepsis is the third most common cause of infant mortality in the world, but its prevalence varies depending on the given region, time and hospital ward (28). For instance, the prevalence of sepsis was 30% in Adib’s study in 2000 but 13.7% in the present study (29). In general, the sampling and detection criteria, the antibiotic history of the mother and infant, the hygiene status of the study population might result in various prevalence rates for septicemia in different regions and times (30). Accurate statistics on the prevalence of this infection, its quick treatment and the reduction of the infant mortality associated with it require a valid monitoring system and the improvement of the available diagnostic methods. Several studies have unanimously declared that umbilical cord blood culture is a simple, painless and reliable approach for diagnosing EOS. Kalatia and Mandot introduced UCB culture as a useful method for the diagnosis of neonatal sepsis with an 80% susceptibility and 91.4% specificity (1). In the present study, UCB infections had a prevalence of 1.85%, and all the neonates with this infection had EOS manifestations. Despite the use of precise diagnostic techniques in this study, such as the BACTEC system, the significant reduction observed in the frequency of this infection might have been caused by the increased awareness among NICU personnel about the importance of compliance with health standards. In a study by Sayehmeri et al. the prevalence of neonatal sepsis was estimated at 14.3% in Iran, which is consistent with the results of the present study (30).

Two UCB cultures were positive in the present study, while the peripheral blood cultures and the sepsis symptoms were negative, which could be caused by the contamination of the UCB samples during the sampling stage. Moreover, a high percentage of the newborns had negative UCB cultures, which shows the lower risk of vertical transmission, probably because of the early diagnosis of the condition and the prophylactic therapies given to the mothers. The present findings showed that EOS has a lower frequency than LOS, which is consistent with the results obtained by Lamia Mohsen and other researchers in Egypt and South Africa (22). The high prevalence of LOS in this study can reflect the importance of hospital sanitation and infection control (hand, catheter and medical equipment hygiene).

Improving infection control and prevention strategies in ICUs and advising physicians about the most common etiologic agents of this infection and their antibiotic resistance are essential measures for ensuring effective therapy and a reduced prevalence of sepsis and its complications in premature infants.

The limitations of the study include the lack of tracing of the genes involved in the resistance mechanisms of Gram-positive strains such as enterococci and staphylococci, especially since they showed greater resistance to antimicrobial agents than the Gram-negative strains in this study. In addition, using techniques such as Whole Genome Sequencing (WGS) can offer a better understanding of the transmission of the pathogenic agents and analysis of the antibiotic resistance of them.

## CONCLUSION

The relatively high prevalence of *bla*_OXA48_ and *bla*_CTX-M15_ genes among bacterial isolates from a life-threatening infection such as neonatal sepsis is a huge concern and the selective pressure resulting from the excessive and empirical use of antibiotics in the community and hospitals leads to the replacement of the susceptible isolates with the resistant. The present study showed that *Enterobacteriaceae* and *A. baumannii* isolates were able to produce broad-spectrum β-lactamase and carbapenemases, respectively. Therefore, careful monitoring is necessary to detect and determine the antibiotic sensitivity of bacterial isolates in order to prevent the spread of these resistant genes among other strains through selecting the proper antimicrobial agent.
